# Somatic transposition in the brain has the potential to influence the biosynthesis of metabolites involved in Parkinson’s disease and schizophrenia

**DOI:** 10.1186/1745-6150-7-41

**Published:** 2012-11-23

**Authors:** György Abrusán

**Affiliations:** 1 Synthetic and Systems Biology Unit, Institute of Biochemistry, Biological Research Centre of the Hungarian Academy of Sciences, Temesváry krt. 62, Szeged H-6701, Hungary

**Keywords:** Retrotransposition, Brain, Metabolic network, Parkinson’s disease, Schizophrenia

## Abstract

**Abstract:**

It has been recently discovered that transposable elements show high activity in the brain of mammals, however, the magnitude of their influence on its functioning is unclear so far. In this paper, I use flux balance analysis to examine the influence of somatic retrotransposition on brain metabolism, and the biosynthesis of its key metabolites, including neurotransmitters. The analysis shows that somatic transposition in the human brain can influence the biosynthesis of more than 250 metabolites, including dopamine, serotonin and glutamate, shows large inter-individual variability in metabolic effects, and may contribute to the development of Parkinson’s disease and schizophrenia.

**Reviewers:**

This article was reviewed by Dr Kenji Kojima (nominated by Dr Jerzy Jurka) and Dr Eugene Koonin.

## Findings

Transposable elements (TEs) make up at least half of the human genome [1,2], however, the vast majority of the approximately 3.5 million TE insertions are fixed, ancient repeats. The overall impact of this enormous number of insertions on the evolution of the genome is still under intensive research; it has been shown that at least 25% of mammalian promoters contain a transposable element [3], a number of genes and protein domains were derived from transposable elements [1,4,5], the presence of TEs can result in alternative splicing [6,7] and structural variation of the genome [8]. Other significant effects of TEs include the inactivation of the X chromosome [9,10], or the modification of gene expression patterns [11].

Despite the very high number of fixed insertions, the number of transpositionally active TEs in the human genome is surprisingly small. The only active autonomous transposon is the L1 retrotransposon, with approximately 80–100 copies contributing the majority of retrotransposition events in the genome [12]. In primates L1s are parasitized by Alu and SVA elements: TEs which do not encode their own proteins, but hijack the proteins of L1s for retrotransposition, and which (Alus in particular) generated very high number of insertions during the evolution of primates. Although the millions of fixed insertions in our genome have mostly neutral, nearly neutral or even beneficial effect on fitness, the recent, polymorphic insertions of active TEs are likely to be harmful, and were shown to be responsible for a number of diseases, including hemophilia, leukemia, colon cancer or breast cancer [13]. In order to reduce their negative fitness effect on the host, TEs were assumed to “jump” only in the germline, as somatic insertions are not inherited, and may seriously harm the host. This view was challenged by recent findings which show that the rate of somatic transposition is higher than expected [14,15]. Particularly high level of somatic transposition was detected in the human brain [16-18], which involves all three types of the active human retrotransposons [16], and is ongoing in several regions of the brain, like hippocampus, middle temporal gyrus or caudate nucleus.

Currently it is unknown how the observed pervasive transposition in the brain influences its functioning, whether it is responsible for any pathological processes, and why neural tissue is so permissive for retrotransposition. Several authors [17,19,20] proposed that, since somatic transposition results in differences between individuals, it may significantly contribute to the cognitive and behavioral differences between humans. On the other hand, since TE transposition in the brain targets a large number of genes [16] it may also contribute to various ageing-related neurological disorders.

In this paper, I computationally investigate what effect somatic transposition can have on the metabolism of the brain, and the biosynthesis of its key metabolites, like neurotransmitters. TE activity in the brain targets a large number of genes, and can significantly influence their functioning, either by disrupting an exon, or through intronic insertions which can also greatly reduce the expression of a gene [11]. However, cellular networks in general are quite robust against perturbations, and, as a consequence, the reduction of expression or even knockdown of a particular gene may not have any phenotypic effects, either because the affected metabolites can be synthesized through alternative pathways, or due to the presence of isoenzymes. In consequence, it is necessary to consider the behavior of the entire metabolic network when analyzing the effects of individual insertions. The analysis shows that the activity of TEs can influence the biosynthesis of a number of neurotransmitters like dopamine, serotonin or glutamate, and may contribute to several diseases of the brain, particularly Parkinson’s disease and schizophrenia.

### Estimating the effect of reduced gene expression on the biosynthesis of metabolites

The insertion of a TE into an exon typically results in a gene knockout, however the majority of TE insertions identified by Baillie et al. [16] inserted into introns. Intronic insertions of L1 retrotransposons, especially in the sense orientation can result in significant, ~70-fold reduction of the expression level of the gene [11], due to the poor transcriptional elongation of the ORF2 of L1s. I tested whether the reduction of expression in any of the genes of the network that was hit by a TE insertion reported by Bailie et al. [16] has an effect on the biosynthesis of any of the metabolites of the network, using a combination of flux variability and flux balance analysis, that has been shown to predict accurately the phenotypic changes associated with genetic modifications [21,22]. No distinction between the types of inserting TEs were made, because all of them are mobilized by the L1 transpositional machinery, target the same insertion sites, and therefore can influence the same set of genes. Experimental studies indicate that TE insertions affect a very large number of cells in the brain, however within each cell there are likely to be only a few new insertions, therefore I assumed that only a single gene is influenced per cell, and the synergistic effects of multiple TE insertions were not investigated.

First, flux variability analysis was performed using the SurreyFBA tool [23] for each of the 2766 non-boundary metabolites of the human metabolic network, Recon1 [24], using an exchange reaction for each metabolite as an objective function. This allowed establishing the flux rate boundaries of each reaction when the network is used to produce one particular metabolite. Next, I identified the genes which, at least theoretically, can be knocked down in the metabolic network with a metabolic effect – i.e. they have no isozymes. Gene expression levels and flux rates of metabolic networks can be coupled in two basic ways: Covert et al. [25] used a binary approach to link expression with flux – above a certain expression level the flux is “on”, while below it the reaction is essentially knocked down. In contrast Colijn et al. [26] used continuous (e.g. linear) functions to couple expression with flux. I used a linear function to approximate the change in flux rates due to TE insertions, because this way no additional parameters need to be estimated. Han et al. [11] reported that L1s inserted into an intron in a sense orientation caused a 70-fold decline in the expression of the gene, and a similar, 70-fold reduction in the flux of the reactions catalyzed by it was assumed (note that the exact amount of flux change assumed does not qualitatively affect our results). To determine which metabolites are synthesized at a reduced rate due to TE insertions, the change in the production rate of each metabolite of the network was calculated, when the reactions catalyzed by a somatic TE-insertion containing gene had reduced fluxes (70-fold).

### Identification of the metabolites influenced by TE insertions

Altogether, the TEs reported by Baillie et al. [16] inserted into 401 genes of the metabolic network, which catalyze 780 reactions. However, in 217 cases the genes carrying a TE insertion have isoenzymes, thus reducing their expression level, or even knocking them out by a TE has no phenotypic effect. From the remaining 184 genes, more than 91 have no effect individually on the biosynthesis of any metabolite. Only insertions in 93 genes resulted in a change of the biosynthesis of any metabolite (Additional file 1: Table S1, Additional file 2: Figure S1). The change in the expression of these genes influenced the biosynthesis of 256 different metabolites, frequently in several organelles (Additional file 1: Table S1), including key neurotransmitters like dopamine, serotonin and glutamate.

The data provided by Baillie et al. [16] came from three individuals (donors A, B and C), and the comparison of the TE-influenced metabolites show large differences between them. Altogether, there are only 9 metabolites that are influenced by TEs in all three donors (Additional file 1: Table S1), none of which are neurotransmitters or associated with a widespread neurological disease. This small number of overlapping metabolites is mostly due to Donor B, in which TEs altogether can influence the biosynthesis of only 9 metabolites, all of which are also affected in donors A and C. The number of metabolites influenced in the other two donors (A and C) is considerably higher (138 and 210 respectively, Additional file 1: Table S1), and also the overlap between them is higher (69 metabolites, Additional file 1: Table S1). The identification of metabolites which are consistently influenced by TE activity in most humans would require data from much more donors, nevertheless the large variability seen in these three individuals already highlights the very large differences in the effect TEs can have in different individuals.

### Metabolic fingerprints of diseases influenced by TE insertions

The reduced biosynthesis of neurotransmitters and other metabolites can result in several diseases of the central nervous system (CNS), and also diseases can manifest themselves with characteristic metabolic profiles. I tested whether the metabolites that are influenced by the activity of TEs are associated with diseases of the CNS, using the metabolite – disease associations provided by the Human Metabolome Database (v.2.5) [27]. The pooled data from all three donors show that TE insertions reduce the biosynthesis of several metabolites that are linked to a disease, most importantly metabolites connected with Parkinson’s disease, Alzheimer’s disease, or schizophrenia (Table 1). However, this is partly due to a simple mass-effect: inserting a sufficiently large number of TEs into random genes is likely to result in changes in the biosynthesis of several metabolites, some of which can be expected to be linked to diseases of the CNS. To separate the diseases which may simply be byproducts of random effects (i.e. mostly the magnitude of TE activity) from those that are the results of the target-specificity of transposition, Monte Carlo simulations were performed to determine the expected number of affected metabolites given the number of observed TE insertions. The endonuclease of L1 retrotransposons has a relatively well defined target site preference, and typically nicks DNA at TT|AAAA hexamer [28] and its variants. I first identified the location of possible target sites (TTAAAA hexamers on both strands, allowing for one mismatch) in all 1496 genes of the human metabolic network. Next, the TE insertions were randomly distributed across these target sites 100 000 times, generating random samples. In each random sample, the genes containing the TE insertions, and those metabolites which are synthesized at a reduced rate due to these insertions were determined. Using the Human Metabolome Database [27], I identified the number of metabolites in each sample that are connected to a particular disease, and tested whether the number of such metabolites observed in samples from the brain is significantly higher than the random expectation using the equation: p = ( n + 1 ) / ( N + 1 ), where p is statistical significance, n is the number of random samples with equal or higher number of affected metabolites as in the brain, and N is the total number of random samples (100 000).

**Table 1:** The list of diseases of the central nervous system (CNS) that can be linked to metabolites influenced by TE insertions

**Nr.**	**Disease**	**Expected**	**SD**	**Observed**	**P**	**Metabolites**
1	Parkinson’s disease	2.318	1.734	7	**0.004**	3-Methoxytyramine,Dopamine,Fe2+, Homovanillate,Phenethylamine,Serotonin, Taurine
2	Alzheimer’s disease	6.539	1.433	8	0.244	3,4-Dihydroxy-L-phenylalanine, L-3-Amino-isobutanoate,L-Cystathionine, Dopamine,Fe2+,Succinate,L-Tyrosine, 24-Hydroxycholesterol
3	Schizophrenia	2.447	1.257	5	**0.040**	4-Hydroxyphenylacetate,Dopamine, Homovanillate,Taurine,L-Tyrosine
4	Canavan disease	2.967	1.104	4	0.338	Oxidized glutathione,Reduced glutathione, Orotate,Succinate
5	Autism	0.156	0.377	2	**0.005**	L-Cystathionine,Homovanillate
6	Epilepsy	3.156	1.184	4	0.376	4-Hydroxyphenylacetate,Homovanillate, Taurine,L-Tyrosine
7	Friedreich’s ataxia	0.222	0.443	1	0.210	Homovanillate
8	Major depressive disorder	0.636	0.589	2	0.057	Homovanillate,Serotonin
9	Multiple sclerosis	3.458	1.489	5	0.224	Cholesterol,Fe2+,Oxidized glutathione, Reduced glutathione,24-Hydroxycholesterol
*10*	Hypothyroidism	3.100	1.484	6	**0.027**	Dopamine,Homovanillate,Serotonin, L-Thyroxine,Triiodothyronine,L-Tyrosine

The expected numbers of such metabolites (+/− SD), and P were estimated by Monte Carlo simulations, which show how many such metabolites would be expected if the same number of TE insertions as observed by Baillie et al. (16) were distributed across the genes of the human metabolic network randomly. Hypothyroidism is also included, because it shares metabolites with several diseases of the CNS, like dopamine, serotonin, homovanillate or L-Tyrosine.

**Figure 1:**
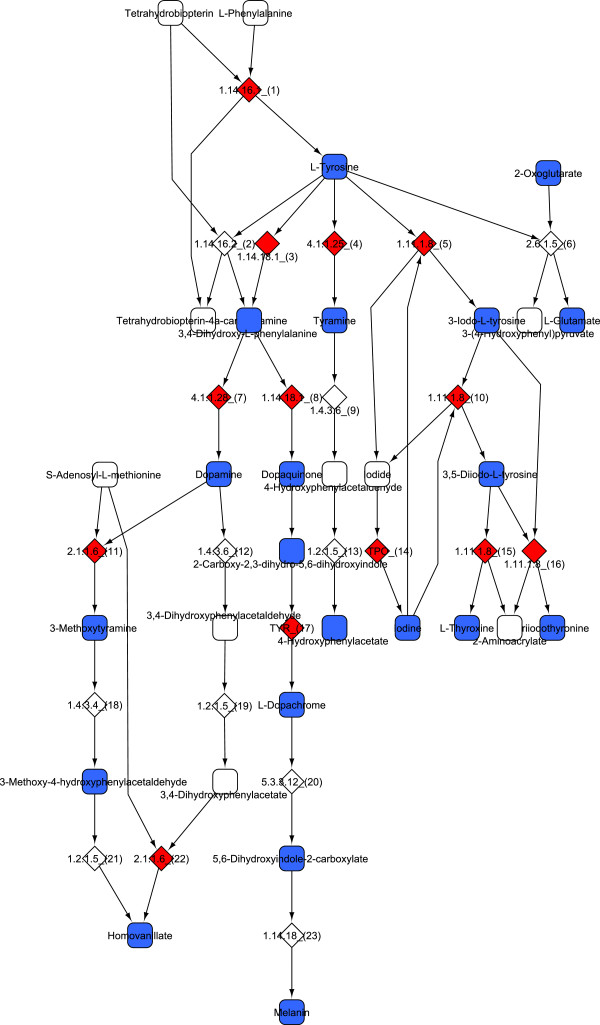
**The pathways of tyrosine metabolism which are most influenced by somatic transposition in the brain.** Reactions with their EC numbers or catalyzing gene are represented with diamonds, metabolites with squares. Reactions which are influenced by TE insertions are highlighted with red; metabolites, which in consequence can be produced only at reduced rate are highlighted with blue. These include key metabolites like tyrosine, dopamine, glutamate, homovanillate or melanin.

The results show that the number of metabolites influenced by TEs is significantly higher than expected by chance in the case of Parkinson’s disease and schizophrenia, and also autism, although in the latter only very few metabolites are involved (Table 1). A large fraction of TE-influenced metabolites that could be linked to a disease of the CNS is synthesized in the tyrosine metabolic pathway, like dopamine, homovanillate, 3-metoxytyramine, tyrosine or 4-hydroxyphenylacetate (Figure 1), but also other key metabolites like glutamate or melanin. Individual donors however, although show an enrichment of disease linked metabolites for most diseases of the CNS (Additional file 1: Table S2), do not show significant effects, which is consistent with the donors being healthy individuals.

### Possible contribution of TE activity to the development of neurological disorders

The results indicate that TE activity in the brain can influence the biosynthesis of several metabolites, including key neurotransmitters like dopamine, serotonin and glutamate, and that Parkinson’s disease (PD) in particular, but also schizophrenia (SZ) and autism are the prime candidates for diseases where the somatic activity of TEs can contribute to the causes. TEs may contribute to these diseases in different ways. First, continuous transposition into genes responsible for, for example tyrosine metabolism can gradually reduce the brain’s ability to synthesize dopamine and related metabolites (Figure 1, Table 1), which may result both in its lowered overall levels, and regional differences within the brain. As TEs are known to be mobilized by environmental stressors [29-31], the magnitude of this process may be significantly influenced by the environment of individuals, which is consistent with the observed large differences between the three donors. Second, transposition may simply result in the loss of neurons, and thus regional differences in TE activity can also result in the faster deterioration of the tissues more prone for transposition.

Our current knowledge on these diseases supports these hypotheses. In the case of PD the underlying cause is unknown, its heritability is low [32,33], the susceptibility loci identified by genome-wide association studies [34] explain only a fraction of the cases, and its onset is thought to be mostly due to environmental factors [35]. The main symptoms of PD are caused by the reduction of dopamine production in the brain, particularly by the gradual decay of dopaminerg cells in the substantia nigra [35]. The case of SZ is probably more complex, as several disorders (psychoses, manias, SZ) form a continuum of symptoms, and may have different physiological causes. Unlike in PD the heritability of SZ is high [36,37], however it is inherited in a non-Mendelian manner and it has proved to be remarkably difficult to identify the susceptibility loci [37,38]; the current view is that thousands of loci may contribute to the disease [37]. However, dopamine (and in consequence tyrosine metabolism) is also thought to have a critical role in the development of symptoms, although in a more complex way than in the case of PD, as the amount and sensitivity for dopamine in SZ patients shows regional differences in the brain: deficiency in the prefrontal cortex and hypersensitivity of dopamine receptors in the mesolimbic pathways; it is likely that the dysregulation of dopamine levels is the primary cause of the disease [39].

Although major depressive disorder (MDD) shows only borderline significance (p = 0.057), the possible influence of TEs on serotonin levels suggests that TEs might be involved to a certain degree. The heritability of MDD is intermediately high, 37% [40]; its causes are largely environmental, stress related, and most likely linked to the loss of neurons in the limbic structures of the brain, e.g. hippocampus [41].

Since the frequency of these diseases in the population is relatively low (1-2% for PD and SZ, 10% for MDD), and all three donors were individuals with no signs of neurodegenerative diseases [16], one should not expect the signatures of full-blown diseases in this dataset, rather indications pointing towards such conditions. I hypothesize that, similarly to diseases like cancer, where the causative mutations can accumulate for decades before the malignant transformation, a long term accumulation of TE insertions in the brain is necessary for the emergence of these diseases, and that their effects reach the necessary threshold only in a fraction of individuals.

Overall, the analysis shows that the ability of TEs to interfere with the biosynthesis of several metabolites, particularly tyrosine metabolism may contribute to significant neurological disorders, and – unless it provides some key benefits for the CNS – to reduce their incidence it may be necessary to develop drugs that halt somatic transposition in the brain.

## Competing interests

The author declares that no competing interests exist.

## Authors’ contribution

The author has read and approved the final manuscript.

## Reviewers’ comments

Reviewer 1: Dr Kenji Kojima, Genetic Information Research Institute, Mountain View, CA, United States of America (nominated by Dr Jerzy Jurka, Genetic Information Research Institute, Mountain View, CA, United States of America)

The manuscript by Abrusán et al. describes computational analysis of the effects of transposon insertions reported by Baillie et al. 2011. The main point of this manuscript is to demonstrate the potential contribution of transposon insertions to neurological disorders by an analysis taking metabolic networks into account. While the topic is very interesting, and the approach is innovative, there are problems with the analysis, so I recommend that the authors reanalyze the data.

Major points:

The most significant problem is the reliability of the analysis reported at pages 7–8. Target specificity of L1 for TT/AAAA is not high, and most of L1 (and Alu and SVA) are inserted into variants of TT/AAAA. However, in their Monte Carlo simulations, the authors assume that L1/Alu/SVA elements are exclusively inserted into TT/AAAA. Therefore, the expected numbers in these simulations may be unrealistic. There may be significant differences between the real data from the simulations due to non-specific insertions. Potentially, some of the genes causing these diseases may have more sites similar to TTAAAA than expected by chance.

Authors’ response: I relaxed the required target specificity in the randomization protocol, and allowed for one mismatch at any position along the TTAAAA hexamer. This resulted in somewhat less significant enrichments (most notably in the case of major depressive disorder, where p = 0.057, and also for Donor A, Additional file 1 Table S2), but did not change the main conclusions of the manuscript.

The authors assume that insertions of Alu and SVA reduce transcription of inserted genes in the same way as L1. However the 70-fold reduction in transcription of gene inserted by L1 is likely to be L1-specific and not applicable to Alu and SVA insertions.

Authors’ response: Certainly, Alu insertions ale less deleterious than L1s, and it is also less easy to quantify their direct effects caused for example by alternative splicing. I treated all TE insertions similarly, because all of them are mobilized by the L1 transpositional machinery, target the same sites, and I was primarily interested in the genes the can be targeted by L1s. (Basically I assumed that individual insertion sites/insertions are not similar between individuals, but that transposition is constrained to a limited number of genes). Also, I was mostly interested in the qualitative effects on metabolism (i.e. which metabolites are influenced), rather that the quantitative effects (how much flux is reduced), and the conclusions would be equally valid if I would have assumed a 50% (or other) reduction in flux after a TE insertion.

The information in the materials and methods is not detailed enough for a full evaluation. Due to a limited description, it is not clear whether the authors take downstream cascade of the network into account. For example, if the expression of Tyramine is reduced by the transposon insertion the downstream metabolites such as 4-Hydroxyphenylacetaldehyde and 4-Hydroxyphenylacetate should be also reduced.

Authors’ response: In estimating the impact of a gene, our flux balance analysis-based approach takes into account every metabolite influenced by the change of expression of that particular gene within the network, including changes resulting from a downstream cascade.

Quality of written English: Acceptable

Reviewer 2: Dr Eugene Koonin, NCBI, NLM, NIH, United States of America

This is a very straightforward analysis with extremely interesting, provocative results. Somatic transposition is a remarkable, novel phenomenon, and here it is convincingly shown with flux balance analysis that such transpositions could reduce expression of many enzymes and consequently affect biosynthesis of neurotransmitters (among other metabolites). Still, the connection to diseases is fully circumstantial, so although the authors do not hide this, perhaps even more caution is advisable. In particular, I would feel more comfortable with a title like:

“Somatic transposition in the brain has the potential to influence the biosynthesis of metabolites involved in Parkinson’s disease, schizophrenia and depression”

Authors’ response: I changed the title as suggested.

Quality of written English: Acceptable

Reviewers’ comments (after revision)

Reviewer 1: Dr Kenji Kojima, Genetic Information Research Institute, Mountain View, CA, United States of America (nominated by Dr Jerzy Jurka, Genetic Information Research Institute, Mountain View, CA, United States of America)

My concern is the simulation study in order to distinguish random effects from target-specific transposition. The meaning of “target specificity of transposition” in the text is unclear, but I speculate that it does not mean the L1’s target sequence preference but a tendency to be inserted into genes related to diseases. However the author’s simulation is not suitable to distinguish these two effects. The author allowed one mismatch in target TTAAAA hexamer in the null hypothesis, but still it could be distant from the actual integration pattern of L1 (and Alu, SVA). In that case, although tendency of L1 to be inserted into genes related to diseases is not rejected, the statistical significance may suggest different sequence preference of target site selection by L1. Even if the claim is true, the author should propose an explanation for why transposons are accumulated in genes of certain metabolic pathways related to diseases.

Authors’ response: By target specificity I mean insertion site specificity, i.e. the preferential insertion into the TTAAAA hexamer and its variants. No other adjustments were made in the randomization, thus any differences from the random expectation are likely to be caused by a biological process, for example by preferential insertion of TEs into genes that are overexpressed in the brain (which was already noted in the study of Baillie et al. 2011).

Quality of written English: Acceptable

Reviewer 2: Dr Eugene Koonin, NCBI, NLM, NIH, United States of America

I have no further comments regarding the content of the manuscript, my review and the author’s response.

Quality of written English: Acceptable

## Supplementary Material

Additional file 1 Table S1, Table S2. Click here for file

Additional file 2 **Figure S1. The matrix of genes and metabolites that are influenced by TE insertions.** It was assumed that the insertion of a TE results in a 70-fold reduction of the expression and flux of the reactions catalyzed by the gene, which, however, due to compensatory effects in the network may result in a much smaller reduction in the rate of the biosynthesis of the influenced metabolites. Click here for file
