# Supplementary figures and images for: Somatic transposition in the brain has the potential to influence the biosynthesis of metabolites involved in Parkinson’s disease and schizophrenia
Source: Biol Direct. 2012 Nov 23;7:41. doi: 10.1186/1745-6150-7-41 (PMC3534579; doi:10.1186/1745-6150-7-41)

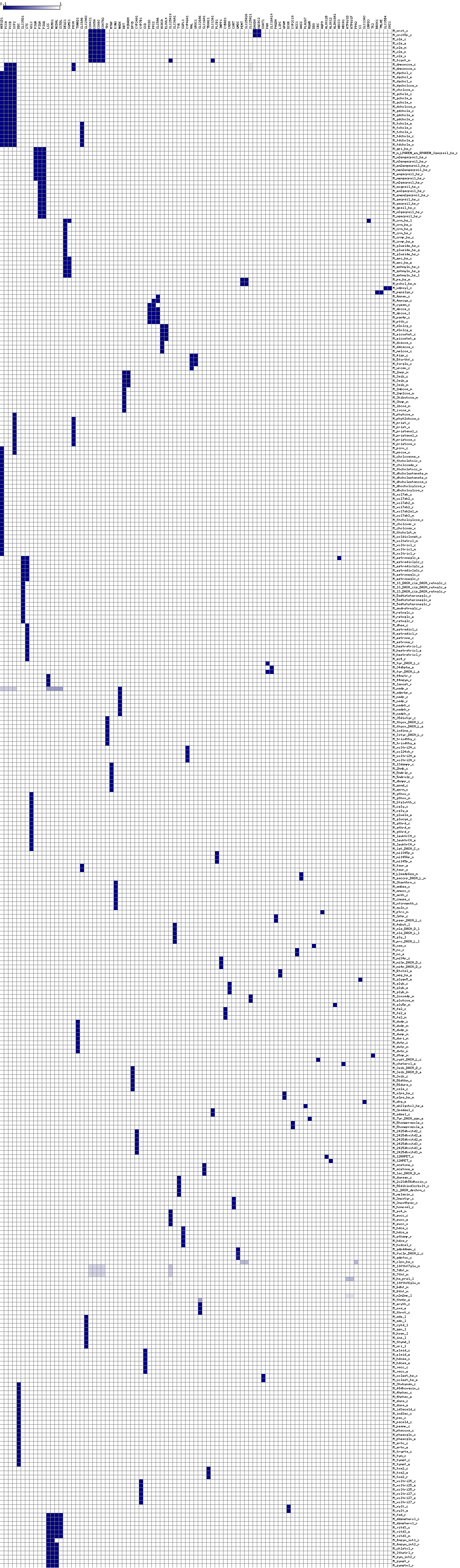

Supplement: Additional file 2 — Figure S1. The matrix of genes and metabolites that are influenced by TE insertions. It was assumed that the insertion of a TE results in a 70-fold reduction of the expression and flux of the reactions catalyzed by the gene, which, however, due to compensatory effects in the network may result in a much smaller reduction in the rate of the biosynthesis of the influenced metabolites. [file 1745-6150-7-41-S2.png]
